# Evolution of Interactions and Cooperation in the Spatial Prisoner's Dilemma Game

**DOI:** 10.1371/journal.pone.0026724

**Published:** 2011-10-31

**Authors:** Chunyan Zhang, Jianlei Zhang, Guangming Xie, Long Wang, Matjaž Perc

**Affiliations:** 1 State Key Laboratory for Turbulence and Complex Systems, College of Engineering, Peking University, Beijing, China; 2 Theoretical Biology Group, Centre for Ecological and Evolutionary Studies, University of Groningen, Kerklaan, The Netherlands; 3 Department of Physics, Faculty of Natural Sciences and Mathematics, University of Maribor, Maribor, Slovenia; Hungarian Academy of Sciences, Hungary

## Abstract

We study the evolution of cooperation in the spatial prisoner's dilemma game where players are allowed to establish new interactions with others. By employing a simple coevolutionary rule entailing only two crucial parameters, we find that different selection criteria for the new interaction partners as well as their number vitally affect the outcome of the game. The resolution of the social dilemma is most probable if the selection favors more successful players and if their maximally attainable number is restricted. While the preferential selection of the best players promotes cooperation irrespective of game parametrization, the optimal number of new interactions depends somewhat on the temptation to defect. Our findings reveal that the “making of new friends” may be an important activity for the successful evolution of cooperation, but also that partners must be selected carefully and their number limited.

## Introduction

Social dilemmas are situations in which the optimal decision for an individual is not optimal, or is even harmful, for the society as a whole. Rational agents, who seek to maximize their own wellbeing, may thus attempt to free ride and reap undeserved rewards, *i.e.* benefit from the “social” contributions of others without providing their own in exchange. However, many simple as well as complex organisms, including higher mammals and humans, exhibit a large tendency towards altruistic behavior. Resolving a social dilemma entails providing a rationale on how can behavior that is costly for an individual but beneficial for the society be maintained by means of natural selection? Achieving a satisfactory understanding of the evolution of cooperation in situations constituting a social dilemma is in fact fundamental for elucidating and properly comprehending several key issues that humanity is faced with today, including sustainable management of environmental resources and warranting satisfactory social benefits for all involved, to name but a few.

Evolutionary game theory has a long and very fruitful history when it comes to understanding the emergence and sustainability of cooperative behavior amongst selfish and unrelated individuals at different levels of organization. Several comprehensive books [1]–[7] and reviews [8]–[12] are available that document the basics as well as past advances in a cohesive and readily accessible manner. The prisoner's dilemma game in particular is frequently employed for studying the evolution of cooperative behavior among selfish individuals. In it's original form, the prisoner's dilemma game consists of two players who have to decide simultaneously whether they wish to cooperate or to defect. The dilemma is given by the fact that although mutual cooperation yields the highest collective payoff, which is equally shared among the two players, individual defectors will do better if the opponent decides to cooperate. Since selfish players are aware of this fact they both decide to defect, whereby none of them gets a profit. Thus, instead of equally sharing the rewarding collective payoff received by mutual cooperation, they end up empty-handed.

A key observation in recent history related to the resolution of the prisoner's dilemma game was that spatial reciprocity can maintain cooperative behavior without any additional assumptions or strategic complexity [13] (see also [14]). Other well known mechanisms promoting cooperation include kin selection [15], direct and indirect reciprocity [16]–[20], as well as group [21], [22] and multilevel selection [23], [24]. These as well as related mechanism for the promotion of cooperation have been comprehensively reviewed in [9]. Another important development that facilitated the understanding of the evolution of cooperation came in the form of replacing the initially employed regular interaction graphs, *e.g.* the square lattice, with more complex networks [25]–[36], whereby in particular the scale-free network has been identified as an excellent host topology for cooperative individuals [37], [38], warranting the best protection against the defectors. Since the strong heterogeneity of the degree distribution of scale-free networks was identified as a key driving force behind flourishing cooperative states [39]–[43], some alternative sources of heterogeneity were also investigated as potential promoters of cooperation with noticeable success. Examples of such approaches include the introduction of preferential selection [44], asymmetry of connections [45], different teaching capabilities [46], heterogeneous influences [47], social diversity [48] as well as diversity of reproduction time scales [49]. Evolutionary games on graphs have recently been comprehensively reviewed in [10], while related coevolutionary games have been reviewed in [12]. Comprehensive reviews concerning complex networks, on the other hand, include [50]–[53].

Coevolutionary games in particular have also received substantial attention recently, for example when studying the coevolution of strategy and structure [54], games on networks subject to random or intentional rewiring procedures [26], [55]–[60], prompt reactions to adverse ties [61], [62], games on growing networks [63], [64], multiadaptive game [65], and indeed many more [66]–[77]. Here we aim to elaborate on this subject further by studying the evolution of cooperation in the prisoner's dilemma game where players are allowed to form new connections with other players that are not in their immediate neighborhoods. Conceptually the study is similar to [78], where it has been reported that the making of new connections promotes cooperation and may help resolve social dilemmas, yet here we focus more precisely on the impact of preference towards linking together more successful players (as opposed to just randomly selecting individuals to connect), as well as on the impact of the number of new links. For this we adopt the linking procedure proposed in [63], but do not allow new players to join, *i.e.* the network does not grow in size. Initially every player is connected only to its four nearest neighbors, and subsequently, at fixed time intervals, 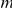 new links are introduced amongst players. Whether more successful players are more likely to receive a new link is determined by a single parameter 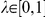, whereby 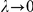 gives all players equal chances (the introduction of new links is independent of the evolutionary success of individuals), while 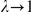 strongly favors the more successful. All the details of the considered setup are described in the Methods section, while here we proceed with presenting the main results.

## Results

**Figure 1:**
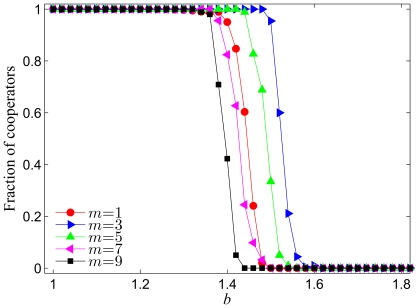
Fraction of cooperators in dependence on the temptation to defect 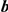 for different values of 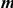. It can be observed that intermediate values of 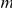 are optimal for the evolution of cooperation, albeit this depends somewhat on the temptation to defect 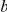. Presented results are averages over 100 independent realizations obtained with the system size 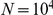 and 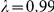. Lines connecting the symbols are just to guide the eye.

We start revealing the properties of the introduced model by examining the impact of the number of newly added links 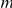 at each full iteration on the fraction of cooperators within the employed prisoner's dilemma game. Figure 1 shows the results obtained by a given combination of the temptation to defect 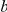 and the parameter 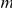. Apparently, the density of cooperators depends strongly on 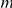. While the fraction of cooperators decreases monotonously from 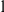 (*i.e.* a state of full cooperation) to 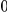 as 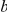 increases, this transition occurs at different values of 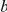 depending on 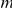. It can be observed that the cooperative behavior is promoted for small and intermediate values of 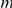, but as the parameter 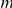 is increased further and exceeds a threshold value (approximately 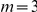), the system undergoes a transition in which the cooperation-facilitative effect deteriorates. These results indicate that an optimal value of 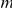 warranting the most significant benefits to cooperators exists. Results presented in Fig. 1 evidence that there exist an optimal amount of new interactions to be added at each full iteration step, determined by 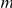 via the coevolutionary process, for which the density of cooperators is enhanced best. It can be argued that for low values of 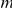 (*e.g.* 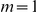 in Fig. 1) the number of newly added links at each iteration is too small to allow the formation of strong hubs, which however, can emerge (see below) if the value of 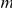 is sufficiently large (*e.g.* 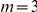 in Fig. 1), yet not too large (*e.g.* 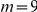 in Fig. 1). It is reasonable to expect that in the optimal case the degree distribution exhibits a heterogeneous outlay (see further below), in particular since such interaction networks are known to promote the evolution of cooperation [37]. Thus, high levels of cooperation are possible even at large 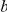, as presented in Fig. 1. However, with 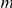 exceeding the optimal value, the chosen players will establish many more connections, too many in fact, thereby essentially reducing the heterogeneity of the resulting interaction network and leaving the whole population in a state characterized by high connectivity resembling well-mixed conditions. Note that in well-mixed populations cooperators cannot survive if 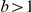, which explains why at large values of 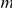 the evolution of cooperation in our case is less successful than at intermediate values of 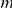.

**Figure 2:**
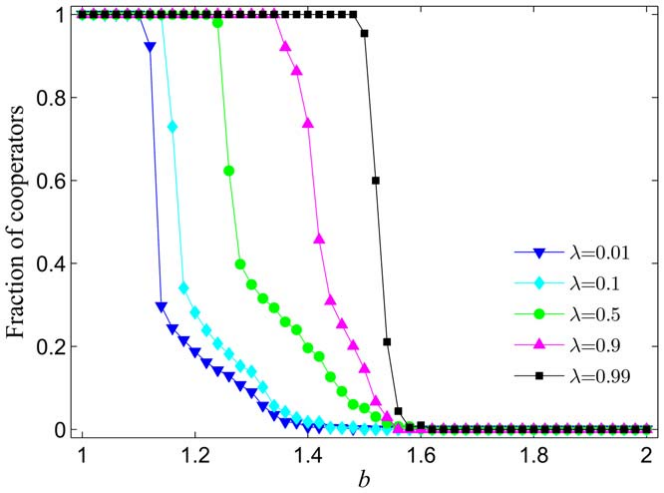
Fraction of cooperators in dependence on the temptation to defect 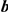 for different values of 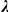. It can be observed that the higher the 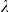 the larger the temptation to defect 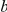 at which cooperators are able to survive when competing against defectors. The span of 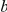 values where cooperators are able to dominate completely increases as well with increasing 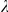. Presented results are averages over 100 independent realizations obtained with the system size 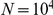 and 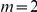. Lines connecting the symbols are just to guide the eye.

The parameter 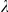 may also significantly affect the outcome of the game. In particular, larger values of 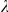 make it more likely for successful players (the ones with high payoffs) to become the recipients of new links. Results in Fig. 2 depict the average level of cooperation 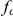 in dependence on the whole relevant span of the temptation to defect 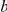 for different values of 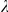. It can be observed that at a fixed value of 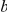 the presently studied model is increasingly more successful by promoting the evolution of cooperation as 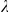 increases. This is somewhat surprising as defectors will be the more successful players at least in the early stages of the game (when there are still enough cooperators to exploit), and thus one could further expect that by obtaining additional links they could outperform cooperators completely. Yet this is not what happens, and indeed when the probability to attach new links to the successful players is large (*e.g.* 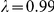 in Fig. 2), the cooperators can remain strong in numbers even if the temptation to defect is high. Based also on previous results [78], it is reasonable to conclude that high values of 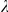 promote the occurrence of a negative feedback effect that is associated with the defective but not with the cooperative behavior. Despite of the fact that initially (in early stages of the game) defectors can successfully extend their base of partners, ultimately their exploitative nature will convert all of them to defectors, and hence there will be nobody left to exploit. Such defector hubs are then quite vulnerable (in terms of the game they are unsuccessful), and are easily overtaken by cooperators. Once cooperators occupy such hubs, their mutually rewarding behavior strengthens their positions quickly, which ultimately paves the way for a successful evolution of cooperation that is here additionally promoted by the coevolutionary process of “making new friends”.

**Figure 3:**
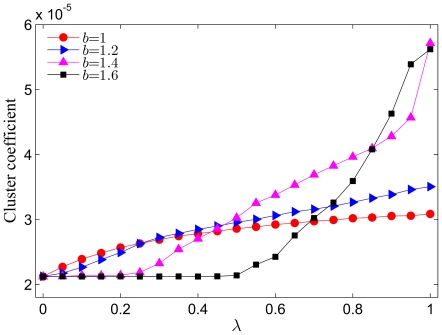
Clustering coefficient of the resulting networks in dependence on 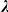 for different values of the temptation to defect 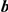. It can be observed that larger values of 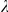 in general lead to more clustered networks, and that higher 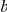 promote clustering as well. Presented results are averages over 100 independent realizations obtained with the system size 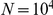 and 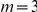. Lines connecting the symbols are just to guide the eye.

Since networks are to be seen as evolving entities that may substantially affect the game dynamics that is taking place on them, it is also important to inspect the degree distribution of players in the employed system for different values of the temptation to defect 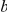 as well as the clustering coefficient associated with the evolved networks. From the results presented in Fig. 3 it follows that the clustering coefficient of the initial square lattice (which is 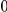) increases due to the addition of new links. This indicates that some realizations (depending on 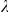 and 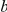) of the coevolutionary game give rise to compact clusters of players. By focusing first on the impact of 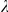, it can be observed that larger values promote clustering, albeit this depends also on the temptation to defect 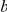. Especially in strongly defection-prone environments the larger values of 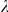 increase the clustering coefficient significantly. Since the parameter 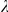 controls the weight (*i.e.* importance) of the payoffs during the coevolutionary process (the addition of new links), these results can be understood well. In particular, for small values of 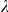 the selection of players that will receive new links is virtually independent of the outcome of the game. In fact, all players are equiprobable recipients of new links, and hence the clustering coefficient is independent of 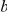. On the other hand, larger values of 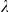 render the selection of the more successful players to become the recipients of new links more likely. From the degree distributions (not shown), we found that larger values of 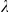 lead to substantially more heterogeneous networks than small 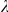. Accordingly, the highly connected nodes are those successful players who accumulate higher payoffs, in turn receiving more and more new links if 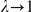. This scenario holds virtually irrespective of 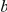, only that for strong temptations to defect the clusters of cooperative players become larger, and accordingly larger is also the clustering coefficient presented in Fig. 3. As is traditionally argued, players located in the interior of such clusters enjoy the benefits of mutual cooperation and are therefore able to survive despite the exploitation from defectors. At this point we can conclude that high values of 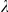 enable cooperative players to grow relatively compact (well clustered) communities starting from their initial nearest neighbors, which in turn strongly promotes the evolution of cooperation, as evidenced by the results presented thus far.

**Figure 4:**
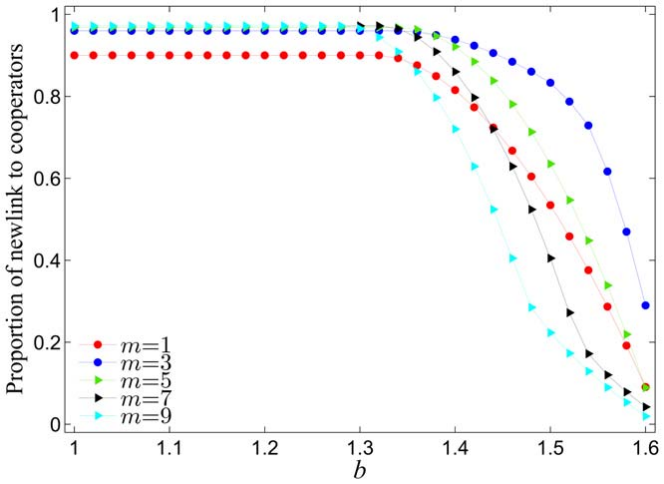
Fraction of new links that are assigned to cooperators in dependence on the temptation to defect 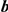 for different values of 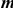. It can be observed that the higher the temptation to defect 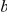, the lower the fraction of new links that are received by cooperators. As by results presented in Fig. 1, it can be concluded that intermediate values of 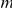 are optimal for cooperators to expand their neighborhoods, although as before, here too this depends somewhat on the temptation to defect 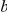. Altogether, this leads to the conclusion that who (either cooperators or defectors) obtains the new links is crucial for the successful evolution of cooperation. Presented results are averages over 100 independent realizations obtained with the system size 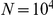 and 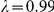. Lines connecting the symbols are just to guide the eye.

**Figure 5:**
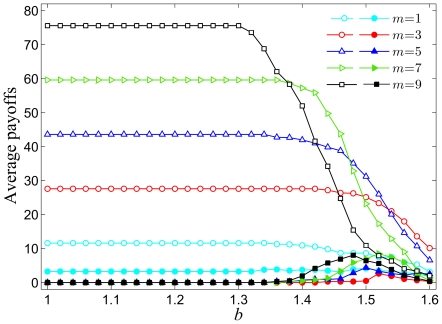
Average payoffs of cooperators (open symbols) and defectors (filled symbols) in dependence on the temptation to defect 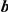 for different values of 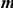. The success of different values of 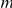 to optimally promote the evolution of cooperation is reflected also in the average payoffs, with intermediate values of 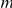 clearly maintaining cooperators more successful than defectors even at high values of 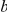. To a lesser extent this is true for small (*e.g.* 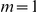) and large (*e.g.* 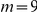) values of 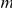, although for small values of 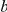 higher values of 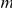 are actually the most effective. The optimal value of 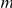 thus depends on the severity of the social dilemma. While low temptations to defect are offset more effectively by larger values of 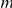, high temptations to defect are dealt with better by intermediate values of 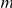 (note that at 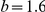 the intermediate value 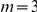 warrants the biggest difference between the average payoffs of the two strategies). Presented results are averages over 100 independent realizations obtained with the system size 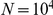 and 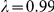. Lines connecting the symbols are just to guide the eye.

With the aim of further enhancing our understanding of the presented results, we investigate this model also from the microscopic point of view, first by showing the fraction of new links received by cooperators in Fig. 4, and second by comparing the average payoffs of cooperators and defectors in Fig. 5. From the results presented in Fig. 4 two regimes can roughly be distinguished. For small values of 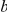 large values of 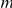 are optimal for cooperators to become the recipients of new links. When going towards larger 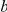, however, there is a crossover, where finally for large temptations to defect intermediate values of 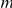 emerge clearly as optimal for cooperators to receive at least some of the “coevolutionary” added links. These observations resonate with the preceding results (see Fig. 1), where indeed intermediate values of 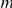 were found to be optimal for the evolution of cooperation, especially at large values of 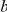. A relative straightforward view into the microscopic workings of the coevolutionary process reveals that this may in fact be because cooperators, despite of their inherent disadvantage over defectors, are still able to acquire at least some fraction of the newly introduced links between players if the value of 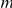 is neither too small nor too large.

Results presented in Fig. 5 lend additional support to those presented in Fig. 4, which is expected since indeed if 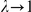 the awarding of new links depends primarily on the payoffs of players. It can be observed that for small values of 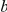 large values of 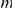 ensure that the average payoff of cooperators is the highest if compared to the average payoff of defectors. When approaching larger 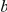, however, there is again a crossover clearly inferable, such that only intermediate values of 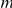 warrant cooperators to outperform defectors in terms of the average payoff. It may come as a surprise that despite of the fact that at 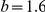 the minority of players is adopting the cooperative strategy (even under optimal conditions in terms of 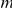 and 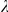) their average payoff is still larger than that of the dominating defectors. After inspecting the distribution of strategies on the network in search for an explanation, we find that even under such unfavorable conditions in small isolated regions of the network the cooperators are surrounded by other cooperators in a very compact manner. Note that the clustering coefficient in this parameter range is relatively large, hence supporting the local formation of such cooperative clusters, in turn warranting a relatively high average payoff for the small population of cooperators. Nevertheless, the cooperators are unable to spread but can only maintain their existence within these clusters that emerge as a sort of a refuge due to the coevolutionary addition of new links, thereby protecting the cooperators from otherwise inevitable extinction.

**Figure 6:**
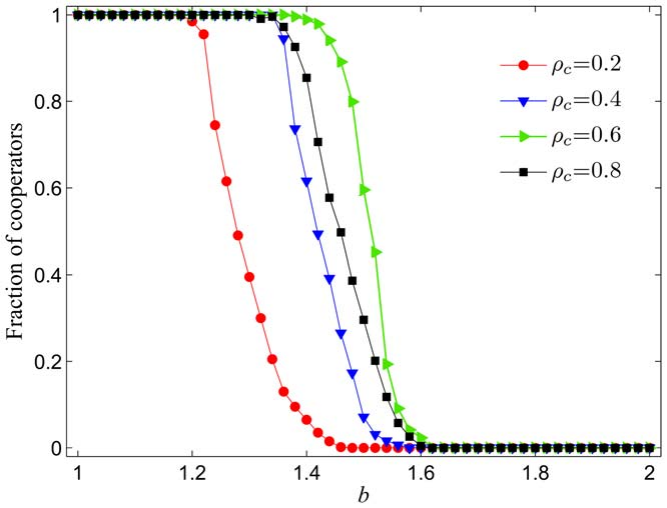
Fraction of cooperators in dependence on the temptation to defect 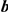 for different initial fractions of cooperators 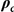. It is interesting to observe that too high initial values of 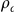 may act detrimental on the evolution of cooperation in the considered model. This may be attributed to the fact that defectors thrive in populations where there are numerous cooperators to exploit, and ultimately this may become a disadvantage in the latter stages of the game. Presented results indicate that an intermediate initial level of cooperators is optimal for the evolution of cooperation. Presented results are averages over 100 independent realizations obtained with the system size 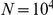, 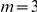 and 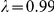. Lines connecting the symbols are just to guide the eye.

Lastly, we also address briefly the issue of the importance of the initial state on the evolution of cooperation in the presently studied model. In Fig. 6 we present the fraction of cooperators in dependence on 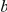 for different values of 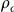, whereby 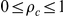 is the fraction of cooperators in the whole population at the beginning of the game. All the results were obtained for 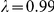, where the addition of new links is driven primarily by the payoff values that the individual players are able to acquire. It is interesting to observe that the initial strategy configuration in the population plays quite an important role. First, it is worth emphasizing the positive aspect, which is that cooperative behavior can ultimately be maintained even when 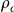 is small (*e.g.* 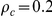 in Fig. 6). Expectedly, for larger values of 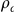 (*e.g.* 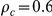 in Fig. 6) the evolution of cooperation is more robust, resulting in complete cooperator dominance over a significantly wider range of the temptation to defect 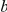. However, with 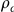 increasing further (*e.g.* 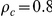 in Fig. 6), the defectors will recapture some advantages, and it becomes obvious that larger values of 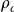 decrease the potentially constructive effect of coevolution on the promotion of cooperation within the present setup. Hence, we arrive at the conclusion that in the long run there is a maximal fraction of cooperators attainable only at an intermediate value of 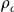.

## Discussion

We have studied the evolution of cooperation in the spatial prisoner's dilemma game where players are allowed to establish new interactions with other players that are not necessarily within their immediate neighborhoods. While the question of whether new links amongst players may potentially promote cooperation has been addressed before [63], [64], [78], we have here reexamined this by focusing more precisely on the impact of preference towards linking together more successful players (as opposed to just randomly selecting individuals to connect), as well as on the impact of the number of new links. In order to achieve this, we have adopted the linking procedure proposed in [63], but did not allow the network of players to grow in size. We have found that the resolution of the social dilemma, here modeled by the prisoner's dilemma game, is most probable if the selection favors the more successful players and if the maximally attainable number of new links added to the population is restricted. More precisely, we have found that the more the selection favors the more successful players, the stronger the promotion of cooperation. Conversely, for the added number of new links it proved optimal if the latter is limited, although this conclusion depends somewhat also on the temptation to defect 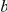. While for low values of 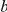 a larger number of new links may be better, for high values of 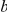 an intermediate number of new links is preferred. We have also examined the dependence of these results on the initial fraction of cooperators in the population, and found rather surprisingly that initially too highly cooperative states are not optimal starting points for the successful evolution of cooperation. We have argued that this may be due to the fact that defectors thrive in populations where there are numerous cooperators to exploit, and ultimately this may become a disadvantage in the latter stages of the game, although this observation may require additional research in order to be better understood. Altogether, our results indicate that new links amongst players may promote cooperation, although it is important to take into account many factors for this conclusion to remain valid. Most importantly, links should be established preferentially amongst the more successful players and must not be too many. This leads us to the reiteration of the statement from the Abstract of this paper, being that the “making of new friends” may be an important activity for the successful evolution of cooperation, but at the same time, it has to be emphasized that friends must be selected carefully and their number kept within reasonable bounds. We hope that this study will motivate further research on coevolutionary games and promote our understanding of the evolution of cooperation.

## Methods

We consider the spatial prisoner's dilemma game where each player occupies a node on the square lattice of size 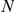 and is connected to its four nearest neighbors. Initially each player is designated either as a cooperator or defector with equal probability unless stated otherwise, and players obtain their payoffs by means of pairwise interactions with all their partners. Following standard practice, the payoffs are 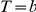 for a defector playing with a cooperator, 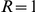 for mutual cooperation, and 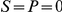 for a cooperator facing a defector and mutual defection, respectively. We thus have the payoff matrix
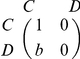
with the only free parameter being the temptation to defect 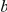. This setup preserve the essential dilemma in that no matter what the opponent does, defection leads to a higher (or at least equal) payoff. Selfish and rational players would therefore always choose defection. But since the payoff for mutual defection is smaller than the payoff for mutual cooperation (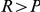) the dilemma arises on what to choose if having in mind also the welfare of the society and not just personal interests. As usual, in one full iteration cycle each agent plays the game once with all its neighbors.

Following payoff accumulation, players attempt to adopt strategies from their neighbors with the aim of increasing their fitness (success) in future rounds of the game. Suppose that player 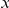 with 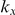 neighbors (initially this will be four, but may increase due to coevolution) accumulates its payoff 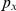. To update its strategy, player 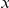 selects one player 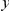 amongst its 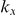 neighbors with equal probability (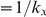). Following [79], we use the Fermi strategy adoption function given by
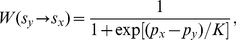
(1)which constitutes the probability that player 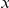 will adopt the strategy of player 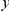, where 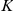 determines the uncertainty by strategy adoptions or its inverse the intensity of selection. In this work we set 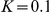, which strongly prefers strategy adoptions from the more successful players, yet it is not impossible that a player performing worse will be adopted either. All the players update their strategies according to this rule in a synchronous manner.

Importantly, here we extend the above traditional setup by allowing players to increase their neighborhoods by linking with players that may be far from their nearest neighbors. Thus, parallel with the evolution of strategies, interactions between players evolve as well. In particular, after every full iteration, 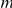 new links are added amongst players while keeping the network size fixed at 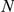. For every new link two individuals are chosen at random from the whole population, with the probability 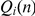 of choosing agent 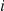 in game round 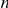 defined as (following [63])
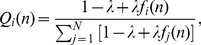
(2)where 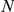 is the system size and 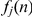 is the accumulated payoff of agent 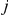. The parameter 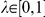 controls the importance of the payoffs in the creation of new links amongst players. The case of 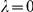 corresponds to neutrality, where each player has equal chances of obtaining a new link, irrespective of its evolutionary success. Conversely, positive values of 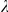 render the selection of the more successful players more likely, *i.e.* players with 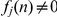 are chosen preferentially, while 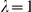 implies that the selection probability is linear with the magnitude of the payoffs (indicating clearly that the most successful players are most likely to obtain new links). We emphasize that self-interactions and duplicate links are omitted. It is also important to note that the continuing addition of new links without growth, *i.e.* new players, evidently leads to a fully connected network. Yet the time scales [80] in this model concerning the evolution of cooperation and the evolution of interactions are very different, such that a quasi stationary state of the two strategies is reached well before full connectedness. Since the focus here is on the evolution of cooperation, we stop the simulations once this quasi stationary state is reached to record the final results.
